# Splenic compensation alleviates impaired-development of bone marrow terminal erythroid to attenuate anemia in ATPIF1 knockout mice

**DOI:** 10.3389/fcell.2025.1675547

**Published:** 2025-10-17

**Authors:** Jing Feng, Yue Zhao, Meiqi Xu, Mengjia Li, Shuchou Xia, Jianping Ye

**Affiliations:** 1 Institute of Trauma and Metabolism, Zhengzhou Central Hospital Affiliated to Zhengzhou University, Zhengzhou, China; 2 Department of Hematology, The First Affiliated Hospital of Zhengzhou University, Zhengzhou, China; 3 Tianjian Laboratory of Advanced Biomedical Sciences, Academy of Medical Sciences, Zhengzhou University, Zhengzhou, China; 4 Zhengzhou Key laboratory of Obesity Research, Zhengzhou Central Hospital Affiliated to Zhengzhou University, Zhengzhou, China

**Keywords:** ATPIF1, mitochondria, erythroid development, proliferation, heme

## Abstract

ATPIF1 (ATPase Inhibitory Factor 1) is a critical regulatory factor of mitochondrial ATP synthase, maintaining ATP homeostasis by modulating ATP synthesis and hydrolysis. In this study, we investigated the consequences of ATPIF1 knockout (KO) on terminal erythroid development and mitochondrial metabolic adaptation in mice. ATPIF1-KO mice exhibited significant reductions in peripheral red blood cell (RBC) counts, hemoglobin, and hematocrit. Mechanistic studies identified impaired development of bone marrow (BM) erythroid, accompanied by robust compensatory erythroid development in the spleen. Integrated RNA-seq and metabolomic analyses revealed that ATPIF1 deficiency disrupted cell proliferation and mitochondrial function in oxidative phosphorylation (OXPHOS) and the tricarboxylic acid (TCA) cycle of BM erythroblasts, leading to defective terminal differentiation of erythrocytes. BM-derived erythroid cells showed a reduction in proliferation, mitochondrial mass, and reactive oxygen species (ROS) levels with an increase in apoptosis. Conversely, the spleen displayed extramedullary erythroid development characterized by enhanced proliferation, reduced apoptosis, increased reductive stress, and upregulation of heme-related genes. Heme levels were decreased in the bone marrow, but not in the spleen. These findings establish ATPIF1 as a key regulator of terminal erythroid development and highlight the essential compensatory role of the spleen in maintaining erythropoietic homeostasis under KO-induced mitochondrial dysfunction. Our work provides new insight into the pathophysiology of mitochondrial-related anemias and potential therapeutic targets.

## Highlights

- ATPIF1 knockout disrupts terminal erythroid development in the bone marrow, triggering compensatory erythroid development in the spleen.
- Mitochondrial dysfunction and metabolic imbalance in ATPIF1-deficient erythrocytes impair proliferation and increase apoptosis.

## Introduction

Terminal erythroid development is a highly regulated process involving the proliferation and differentiation of hematopoietic progenitors into mature RBCs (Palis, 2014). In mammals, this process begins with proerythroblasts that undergo three mitotic divisions, sequentially generating basophilic, polychromatic, and orthochromatic erythroblasts before enucleating to form reticulocytes (Ji et al., 2011). These developmental transitions require massive metabolic resources, with erythroid precursors shifting from glycolysis to mitochondrial OXPHOS to meet energy demands (Lepelley and Crow, 2021). In mitochondria, ATP production via the TCA cycle, OXPHOS and heme biosynthesis are essential for erythroid cell function (Ponka, 1997; Oburoglu et al., 2016; Liu et al., 2017; Richard et al., 2019).

ATPIF1 functions as a critical regulator of F_1_F_o_-ATP synthase (Complex V), modulating the synthesis and hydrolysis function of Complex V (Zhong et al., 2022). By finely tuning these dual functions, ATPIF1 ensures the maintenance of intracellular ATP homeostasis (Zhong et al., 2022). Beyond its role in OXPHOS regulation, ATPIF1 contributes to mitochondrial ROS production under normoxia (Gore et al., 2022; da Silva et al., 2024). Under hypoxic stress, its regulatory influence on the mitochondrial proton gradient becomes especially vital for sustaining ATP synthase activity (Zhong et al., 2023; Ge et al., 2024). Genetic deletion of ATPIF1 disrupts this balance, leading to proton gradient collapse, mitochondrial dysfunction, and cellular energy crisis (Acin-Perez et al., 2023).

The impact of ATPIF1 genetic disruption on heme biosynthesis during erythroid development appears to vary between mammalian and non-mammalian species (Yien and Perfetto, 2022). For instance, ATPIF1 knockout zebrafish exhibit anemia from heme synthesis defects (Shah et al., 2012), while ATPIF1-depleted human CD34^+^ cells and murine erythroleukemia (MEL) cells show impaired erythroid differentiation (Shah et al., 2012). However, our previous investigations revealed that ATPIF1-knockout mice did not develop severe anemia, suggesting the existence of compensatory mechanisms in mammalian hematopoiesis (Nakamura et al., 2013; Zhong et al., 2023).

In this study, we demonstrate that ATPIF1-KO mice exhibit mild anemia characterized by reduced peripheral RBCs and lower hemoglobin levels. BM analyses revealed defective terminal erythroid differentiation, characterized by diminished mitochondrial mass, attenuated ROS production, and increased apoptotic rates among erythroid precursors. Transcriptomic and metabolomic profiling identified impaired proliferation, downregulate of TCA cycle and OXPHOS activity. Compensatory splenic erythroid development in ATPIF1-KO mice featured metabolic reprogramming and cell proliferation. ATP/ADP, lactate/pyruvate and malate/oxaloacetate ratios demonstrated the presence of reductive stress in the spleen (Xiao and Loscalzo, 2020). These findings establish ATPIF1 as a master regulator of mitochondrial metabolism during erythroid development and reveal potential therapeutic targets for mitochondrial-related anemias.

## Materials and methods

### ATPIF1-KO mice

ATPIF1-KO mice on a C57BL/6 genetic background were generated and characterized as previously reported (Zhong et al., 2022). All animals were bred and maintained in the animal facility at Zhengzhou University under standard conditions (20 °C ± 2 °C, 60% ± 5% humidity, 12-h light/dark cycle) with *ad libitum* access to food and water. All experimental procedures involving animals were approved by the Life Sciences Ethics Review Committee of Zhengzhou Central Hospital, affiliated with Zhengzhou University (Approval No. ZXYY2024131). Male ATPIF1-KO mice aged 8–12 weeks were used for experiments, with age-matched wild-type littermates serving as controls.

### Hematology analysis

Peripheral blood counts were measured using a Myriad Veterinary Automatic Blood Cell Analyzer Model: BC-2800vet.

### BrdU detection

Cell proliferation was evaluated using the FITC-BrdU Cell Proliferation Detection Kit (KeyGEN, Cat# KGA9201-20). Mice received intraperitoneal injections of BrdU (100 mg/kg body weight, dissolved in PBS) and tissue samples were collected for analysis 1 week after administration. DNA denaturation was carried out according to the manufacturer’s instructions prior to BrdU detection.

### Cell isolation and preparation

Bone marrow cells were flushed from tibiae and femora with PBS supplemented with 0.5% BSA and 2 mM EDTA, and then passed through 70 μm cell strainers. Spleen cells were mechanically dissociated in PBS containing 0.5% BSA and filtered through 70 μm strainers.

### Flow staining of terminal erythroid cells

BM and spleen single cell suspensions were counted and 2 million cells were taken from each and stained with TER119- Brilliant Violet 421™ (BV421; Cat# 116233, Biolegend), CD44-APC (Cat# 103012, Biolegend), CD45^−^ APC/Cyanine7 (APC-Cy7; Cat# 157204, Biolegend), CD11b- APC-Cy7 (Cat# 101226, Biolegend), and Ly-6G/Ly-6C (Gr-1)- APC-Cy7 (Cat# 108423, Biolegend),APC anti-mouse TER-119/Erythroid Cells Antibody (APC; Cat# 116212, Biolegend). Data acquisition was performed on a BD LSRFortessa SORP flow cytometer, and data were analyzed using FlowJo software (version 10.0).

### Mitochondrial assay

After flow staining of the erythroid cells, cells were incubated with 100 nM Mito Tracker Green (Cat# C1048, Beyotime) at 37 °C for 30 min. Stained cells were washed to remove the Mito-Tracker Green staining working fluid, fresh cell culture medium (pre-warmed at 37 °C) was added, cells were resuspended in fresh cell culture medium (pre-warmed to 37 °C) and immediately subjected to flow cytometric analysis to assess mitochondrial mass, as indicated by MitoTracker Green fluorescence intensity.

### ROS assay

Intracellular ROS levels were measured using the Reactive Oxygen Species Assay Kit (Cat# CA1410, Solarbio). After flow staining, cells were collected and resuspended in serum-free medium containing 10 mM DCFH-DA, a fluorescent probe for ROS detection, and incubated at 37 °C for 20 min. Cells were then washed three times with PBS to remove any extracellular DCFH-DA. After resuspension, ROS fluorescence intensity was immediately measured by flow cytometry.

### Apoptosis detection

After flow staining of the erythroid cells, the apoptosis rate was assessed using the Annexin V-FITC Apoptosis Detection Kit (Cat# C1062, Beyotime). According to the manufacturer’s instructions, cells were collected and resuspended in 195 µL of binding buffer. Followed by addition of 5 μL Annexin V-FITC and 10 μL propidium iodide (PI) solution., Samples were incubated for 15 min at room temperature (20 °C–25 °C) in the dark and subsequently analyzed by flow cytometry immediately. Cell populations were classified as viable (Annexin V^−^/PI^−^), early apoptotic (Annexin V^+^/PI^−^), late apoptotic/necrotic (Annexin V^+^/PI^+^), or necrotic (Annexin V^−^/PI^+^), and the proportion of apoptotic cells was compared between groups.

### Detection of intracellular ferrous iron (Fe^2+^)

After flow staining of the erythroid cells, intracellular Fe^2+^ levels were detected using an Iron Assay Kit (abs47038990; Absin, China). Cells were incubated with 1 µM FerroOrange working solution at 37 °C under 5% CO_2_ for 30 min. After incubation, the cells were washed once with PBS, and the fluorescence intensity reflecting ferrous iron content was immediately analyzed by flow cytometry.

### Heme detection

Single-cell suspensions were prepared from the spleen and bone marrow (n = 6). 5 million cells from each tissue were homogenized by grinding. The homogenates were centrifuged to obtain the supernatant. Spleen supernatant, bone marrow supernatant (diluted to an appropriate concentration), standards, and distilled water were aliquoted into separate wells of a 96-well plate. Reaction Buffer was then added to each well, followed by the addition of the dye reagent. The plate was gently shaken to ensure thorough mixing and incubated at room temperature for 10 min. Absorbance was measured at 505 nm for quantitative analysis.

### RNA-seq of BM CD45^−^ cells

CD45-negative cells were isolated from BM of mice (n = 2 per group) and preserved in TRIzol reagent (Invitrogen) for RNA extraction. RNA sequencing libraries were prepared and sequenced (by Genesky Biotechnologies Inc., Shanghai, China) following standard protocols. RNA concentration was prepared with a minimum required concentration of 50 pg/μL for a total input amount of ≥400 pg in sequencing. Full-length cDNA was synthesized and amplified from the total RNA using the SMART-seq2 protocol. The final libraries with an average insert size distribution of 300–400 bp, as verified by the Agilent 2,100 Bioanalyzer. Libraries that passed quality control were quantified by Qubit, pooled in equimolar ratios, and sequenced on an Illumina NovaSeq 6,000 using a 2 × 150 bp paired-end strategy. Raw sequencing data (raw reads) in fastq format were subjected to quality control and filtering with the following parameters: Removal of adapter sequences, trimming of bases with a Phred quality score (Q) < 15 at read ends, discarding of reads shorter than 40 bp after processing. Differential expression analysis between groups was performed using the DESeq2 (http://bioconductor.org/packages/release/bioc/html/DESeq2.html) R package.

### RNA-seq of spleen CD45^−^ cells

CD45-negative cells were isolated from mouse spleens (WT:ATPIF1-KO = 4:5) and preserved in TRIzol reagent (Magen) for RNA extraction. RNA-seq analysis was conducted by Shanghai Applied Protein Technology (Shanghai). RNA samples were used for subsequent library preparation and sequenced on MGISEQ-T7 platform to generate 150 bp paired-end reads. Raw data in fastq format were initially processed to remove low-quality reads and adapter sequences. The resulting high-quality data are referred to as clean reads. Differential expression analysis between groups was performed using the DESeq2 (http://bioconductor.org/packages/release/bioc/html/DESeq2.html) R package. Genes with an absolute value of log2 fold change (log2FC) > 1 and an adjusted p-value (padj) < 0.05 were considered statistically significant differentially expressed genes (DEGs).

### Targeted metabolomics analysis

Bone marrow and spleen tissues were rapidly harvested and stored in −80 °C freezer after flash-frozen in liquid nitrogen (n = 6 per group). The samples were thawed at 4 °C and 80 mg of each sample was mixed with 25 μL isotope internal standards and 400 μL of cold methanol/acetonitrile solution (1:1, v/v). The lysate was homogenized by MP homogenizer (20s, thrice), adequately vortex, then ultrasounded for 5 min at low temperature, followed by incubation at −20 °C for 1 h. The mixture was centrifuged for 20 min (14000rcf, 4 °C). The supernatant was taken from the Ostro plate, and divided into two tubes (one tube is 2/3 and the other tube is 1/3). 2/3 of the solution was dried in a vacuum centrifuge, re-dissolved in 150 μL acetonitrile/water (1:1, v/v) and adequately vortexed, and then centrifuged (14000rcf, 4 °C, 15 min). The supernatant was then injected into the HPLC-MS/MS apparatus for quantification and analysis by the Applied Protein Technology Company (Shanghai, China).

### Statistical analysis

Unless otherwise specified, all quantitative data are presented as mean ± standard deviation (SD). Statistical significance between groups was evaluated using an unpaired two-tailed Student’s t-test. Sample sizes are indicated in the corresponding figure legends. Flow cytometry data were acquired on an LSRFortessa instrument and analyzed using FACSDiva software (BD Biosciences) and FlowJo v10 (Tree Star, United States). All statistical analyses and graphing were performed using GraphPad Prism. A p-value <0.05 was considered statistically significant. RNA-seq data are presented after FDR (False discovery rate) adjustment and metabolomics data are presented without FDR adjustment.

## Results

### Loss of ATPIF1 impairs BM terminal erythroid development

**FIGURE 1:**
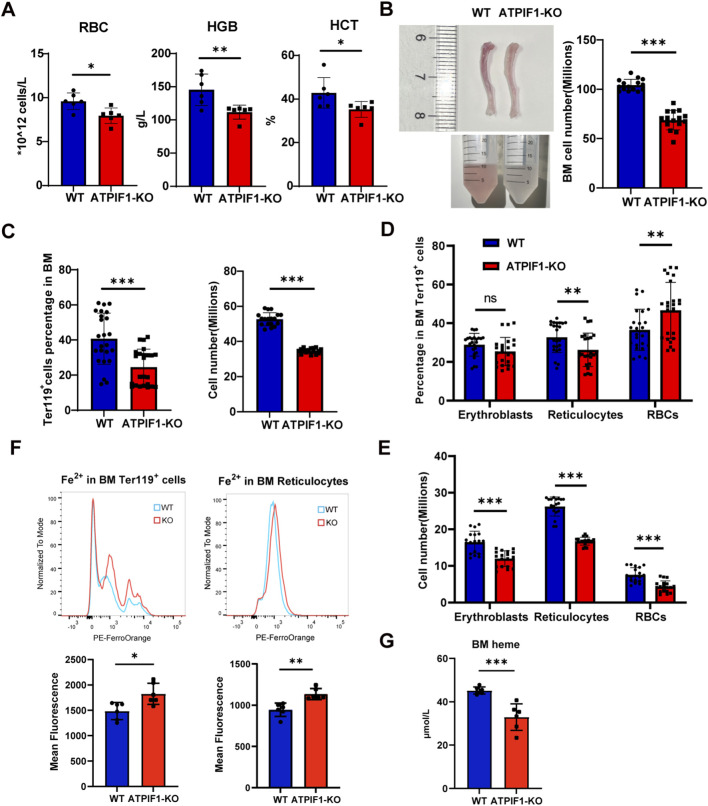
ATPIF1 deficiency impairs BM erythropoiesis in mice. **(A)** Analysis of peripheral blood parameters from 2 to 3 months old WT and ATPIF1-KO mice (n = 6). **(B)** Representative images of bone marrow and corresponding cell suspensions, along with quantification of total bone marrow cell counts (n = 24). **(C)** Quantification and cell numbers of total Ter119^+^ cells (n = 24). **(D)** Quantification of erythroblasts, reticulocytes and RBCs (n = 24). **(E)** Cell numbers of erythroblasts, reticulocytes and RBCs (n = 24). **(F)** Flow cytometric analysis of Fe^2+^ content showing increased mean fluorescence intensity (MFI) in Ter119^+^ cells and reticulocytes in the bone marrow (n = 6). **(G)** Heme level in bone marrow cells (n = 6). Data are presented as mean ± SD; *p < 0.05; **p < 0.01; ***p < 0.001 by unpaired two-tailed t-test.

To elucidate the impact of ATPIF1 deficiency on erythroid development, we evaluated peripheral blood parameters and BM erythroid populations in ATPIF1-KO mice. Blood parameters analysis revealed decreases in RBCs, hemoglobin (HGB), and hematocrit (HCT) (Figure 1A and Supplementary table), while mean corpuscular hemoglobin (MCH) values remained unchanged (Supplementary Figure S1A). Macroscopically, bones from ATPIF1-KO mice appeared noticeably paler compared to wild type mice, and a marked reduction in erythroid cells was observed in BM single-cell suspensions, as indicated by the lighter color of cell pellets (Figure 1B). Typically, a combination of Ter119, CD44, and cell size is utilized to distinguish different stages of terminal erythroid cells in mice by flow cytometry (Liu et al., 2011; Liu et al., 2013). Flow cytometric analysis demonstrated a significant reduction in the proportion and cell numbers of Ter119^+^ cells in the BM of ATPIF1-KO mice. Furthermore, we analyzed the distribution of erythroblasts, reticulocytes, and RBCs during terminal erythroid development in the BM (Figures 1C–E; Supplementary Figure S1D). FerroOrange-based flow cytometric analysis revealed an increase in Fe^2+^ content within Ter119^+^ cells and reticulocytes in the bone marrow. (Figure 1F). But heme levels in bone marrow cells was decreased (Figure 1G).

Elevated ferrous iron levels coupled with reduced heme content in bone marrow cells indicate impaired heme synthesis. Given that mitochondria serve as the primary site for heme biosynthesis (Dutt et al., 2022), these findings strongly suggest mitochondrial abnormalities in ATPIF1-knockout bone marrow cells. These data established that loss of ATPIF1 impairs terminal erythroid development in the bone marrow, resulting in diminished RBC production. Notably, while Ter119^+^ cells were substantially depleted in ATPIF1-KO BM, the mice maintained near-normal HCT, suggesting activation of compensatory erythroid development.

### Splenomegaly and compensatory erythroid development in ATPIF1-KO mice

**FIGURE 2:**
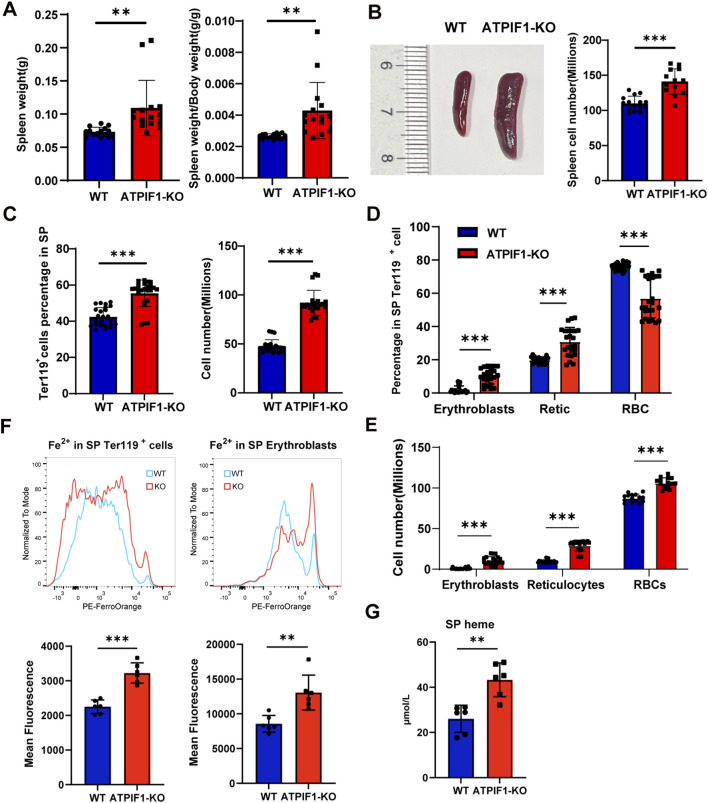
Compensatory splenic erythropoiesis in ATPIF1-KO mice. **(A)** Quantification of spleen weight and spleen/body weight ratio (n = 6). **(B)** Representative macroscopic images of spleens and quantification of total splenic cell count (n = 6). **(C)** Quantification and cell numbers of total Ter119^+^ erythroid cells in the spleen (n = 24). **(D)** Quantification of erythroblasts, reticulocytes and RBCs (n = 24). **(E)** Cell numbers of erythroblasts, reticulocytes and RBCs (n = 24). **(F)** Flow cytometric analysis of Fe^2+^ content showing increased MFI in Ter119^+^ cells and erythroblasts in the spleen (n = 6). **(G)** Heme level in spleen cells (n = 6). Data are presented as mean ± SD; *p < 0.05; **p < 0.01; ***p < 0.001 by unpaired two-tailed t-test.

In line with the compromised terminal erythroid development observed in the BM, ATPIF1-KO mice developed significant splenomegaly while maintaining stable body weight (Figures 2A,B; Supplementary Figure S1B). In contrast to the BM, the spleen of ATPIF1-KO mice exhibited notable expansion of Ter119^+^ populations (Figure 2C; Supplementary Figure S1E), reflecting elevated erythroid precursor cells proliferation and differentiation, which corresponded with an increased proportion and cell numbers of erythroblasts and reticulocytes (Figures 2D,E). Flow cytometric analysis demonstrated an increased Fe^2+^ content in the spleen Ter119^+^ cells and erythroblasts (Figure 2F). And heme level in spleen cells was increased (Figure 1G).

### ATPIF1 deficiency impairs terminal erythroid differentiation of RBC *in vivo*

**FIGURE 3:**
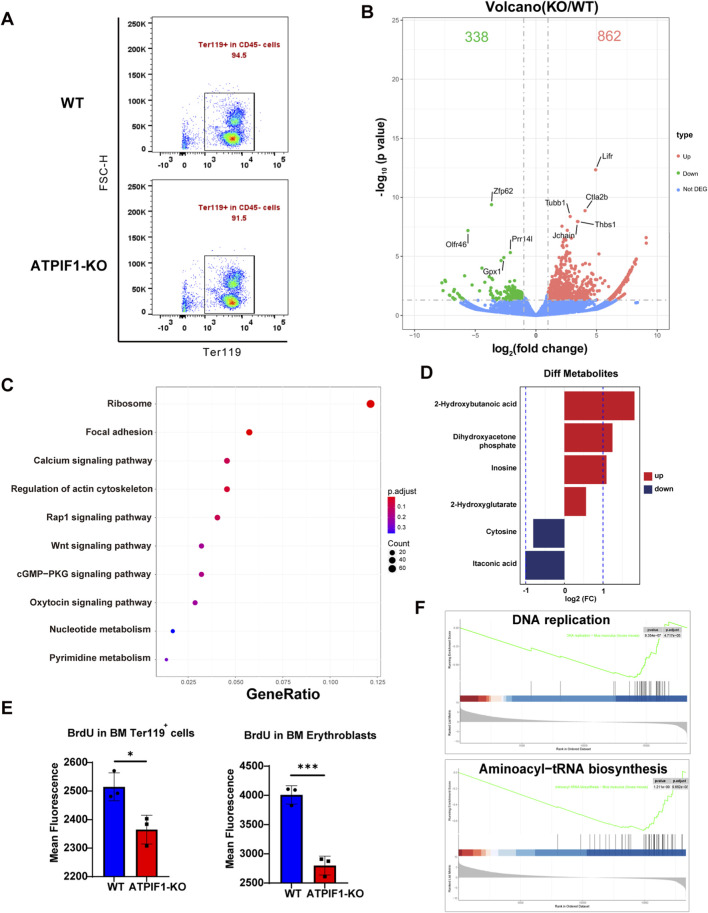
ATPIF1 deficiency impairs the proliferation of BM erythroid cells. **(A)** Proportion of Ter119^+^ cells among CD45^−^ bone marrow cells following magnetic bead sorting. **(B)** Volcano plot of differentially expressed genes (DEGs) in BM CD45^−^ cells from WT and ATPIF1-KO mice. Downregulated (green) and upregulated (red) genes are shown (fold change >2, *p* < 0.05) (n = 2). **(C)** KEGG enrichment scatter plot of DEGs in BM CD45^−^ cells. **(D)** Fold change of significantly altered metabolites in BM from WT and ATPIF1-KO mice BM. **(E)** BrdU incorporation assay showing decreased proliferation, as measured by mean fluorescence intensity in Ter119^+^ cells and erythroblasts (n = 3). Data are presented as mean ± SD; ns > 0.05; *p < 0.05; **p < 0.01; ***p < 0.001 by unpaired two-tailed t-test. **(F)** GSEA of RNA-seq data revealing downregulation of aminoacyl-tRNA biosynthesis pathways and DNA replication pathways in CD45^−^ BM cells, ATPIF1-KO is indicated in blue. NES and FDR-adjusted p values by clusterProfiler are shown.

To elucidate mechanisms underlying the terminal erythropoietic defects in ATPIF1-KO mice, we isolated CD45^−^ cells from BM via magnetic bead sorting and performed RNA-seq. The proportion of Ter119-positive cells among CD45-negative bone marrow cells following magnetic bead sorting exceeded 90% (Figure 3A). Differential expression analysis identified 862 upregulated and 338 downregulated genes in ATPIF1-KO mice (Figure 3B). Untargeted metabolomic profiling uncovered buildup of 2-hydroxybutanoic acid disrupts the NADH/NAD^+^ balance, thereby impairing nucleotide biosynthesis (Sharma et al., 2021); elevated DHAP levels reflect dysregulation of glycolysis and gluconeogenesis (Orozco et al., 2020); inosine accumulation coupled with cytosine depletion indicates compromised purine and pyrimidine metabolism (Abudayyeh et al., 2019). Additionally, increased levels of 2-hydroxyglutarate (2-HG) may be indicative of reductive TCA cycle activity under hypoxic conditions (Munk et al., 2023). Collectively, these metabolic derangements limit both the supply of essential biosynthetic precursors and cellular energy required for proliferation, while reduced itaconate levels suppress SDH, further disrupting TCA cycle function (Selak et al., 2005). ,Downregulation of TCA cycle and OXPHOS pathways, together with decreased ATP/ADP ratios in bone marrow (Supplementary Figure S1D–E), highlight a state of energy deficiency that is unfavorable for cellular proliferation and differentiation (Figure 3D; Supplementary Figure S1D).

Gene set enrichment analysis (GSEA) revealed significant downregulation of genes involved the DNA replication and tRNA biosynthesis pathway pathway (Figures 3C,F). BrdU incorporation assays confirmed significantly reduced proliferation of Ter119^+^ erythroid cells in the BM of ATPIF1-KO mice (Figure 3E), lending functional support to the multi-omics findings. Genetic ablation of ATPIF1 induces systemic dysregulation of energy supply, and redox homeostasis, creating a “metabolic bottleneck” that impairs erythroid development.

### ATPIF1 deficiency disrupts mitochondrial homeostasis and promotes erythroblasts apoptosis in BM

**FIGURE 4:**
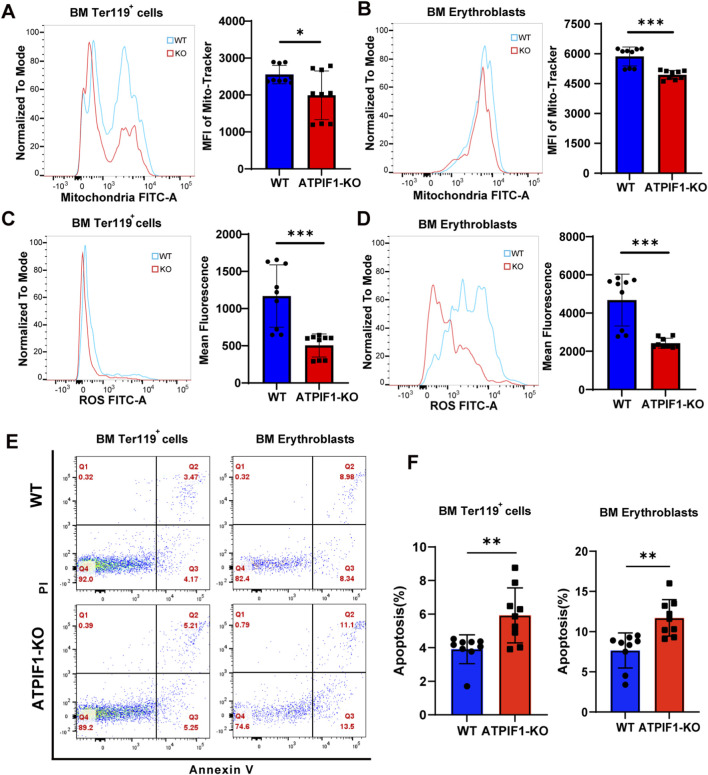
ATPIF1 maintains mitochondrial function and cellular viability in BM erythroid cells. **(A, B)** Flow cytometric analysis of mitochondrial content (MitoTracker Green) showing decreased MFI in **(A)** Ter119^+^ cells and **(B)** erythroblasts from ATPIF1-KO mice compared to WT mice (n = 9). **(C, D)** Intracellular ROS levels measured by DCFDA fluorescence staining in **(C)** Ter119^+^ cells and **(D)** erythroblasts (n = 9). **(E)** Representative flow cytometry plots of Annexin V staining for apoptosis analysis in erythroid populations (n = 9). **(F)** Quantification of apoptosis rates in erythroid cells populations (n = 9). All data are presented as mean ± SD; ns > 0.05; *p < 0.05; **p < 0.01; ***p < 0.001 by unpaired two-tailed t-test.

Mitochondrial content and ROS production were both decreased in BM erythroid cells, correlating with increased rates of apoptosis (Figure 4). Flow cytometric analyses further confirmed decreased mitochondrial content in terminal erythroid cells of the BM (Figures 4A,B), alongside marked attenuation of ROS generation (Figures 4C,D). Apoptosis rates were correspondingly increased in both the Ter119^+^ cells and erythroblasts populations (Figures 4E,F). These findings demonstrate that ATPIF1 deficiency impairs mitochondrial function through coordinated suppression of oxidative metabolism, ultimately triggering apoptosis in BM erythroid progenitors. The observed reduction in both mitochondrial and ROS suggests profound dysregulation of mitochondrial homeostasis following ATPIF1 deletion.

### ATPIF1 knockout promotes compensatory erythroid development in spleen

**FIGURE 5:**
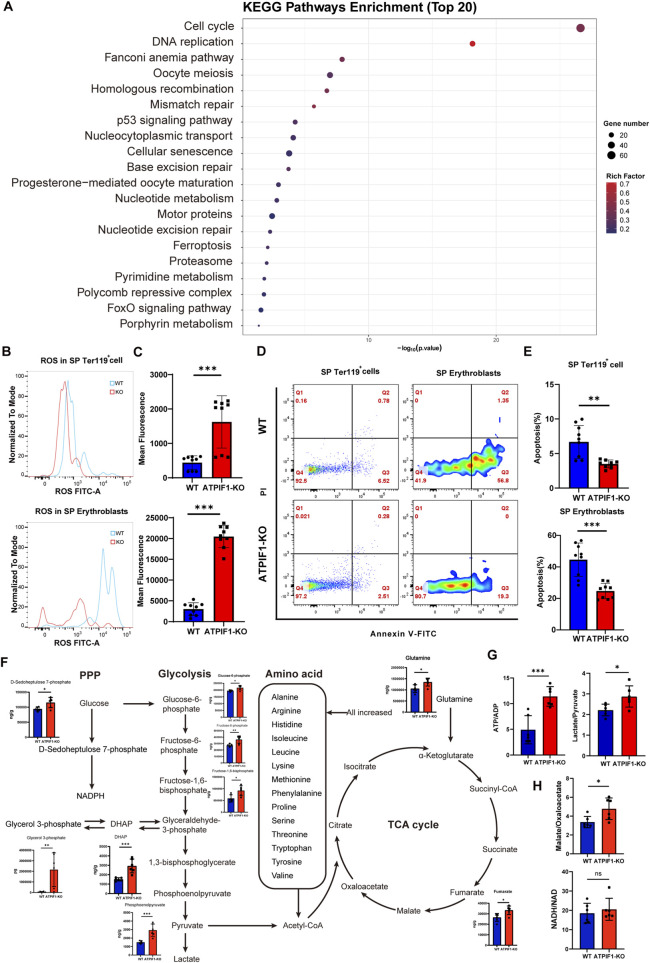
Splenic erythroid adaptations in ATPIF1-KO mice. **(A)** The top 20 enriched Kyoto Encyclopedia of Genes and Genomes (KEGG) pathways identified based on DEGs in splenic CD45^−^ cells. In the pathway plot, circle size corresponds to the number of genes involved, while color intensity reflects the enrichment score (WT:ATPIF1-KO = 4:5). **(B, C)** Flow cytometric analysis of ROS levels in **(B)** splenic Ter119^+^ cells and **(C)** erythroblasts (n = 9). **(D)** Representative flow cytometry analysis of Annexin V staining for apoptosis detection in splenic erythroid cells. **(E)** Quantitative analysis of apoptosis rates in splenic erythroid cells populations (n = 9). **(F)** Alterations of splenic metabolites in glycolysis, TCA cycle, PPP and AAM (n = 6). **(G)** Alterations in ATP/ADP and Lactate/Pyruvate ratios in spleen (n = 6). **(H)** Alterations in Malate/Oxaloacetate and NADH/NAD ratios in spleen (n = 6). Data are presented as mean ± SD; ns > 0.05; *p < 0.05; **p < 0.01; ***p < 0.001 by unpaired two-tailed t-test.

Transcriptomic profiling of splenic CD45^−^ cells isolated from ATPIF1-KO mice demonstrated significant upregulation of cell cycle and DNA replication pathways according to KEGG enrichment (Figure 5A), suggesting enhanced cellular proliferation. These findings are consistent with our compensatory splenic flow cytometry quantification (Figures 2C,D). Importantly, the adaptations observed in the spleen differed from those in the BM, where mitochondrial content was maintained (Supplementary Figure S2A–B), and there was evidence of increased proliferative capacity and improved erythroid cell survival. ROS levels in splenic erythroid cells were markedly increased in ATPIF1-KO mice (Figures 5B,C), and apoptosis rates in splenic erythroid cells were significantly reduced (Figures 5D,E), which is in stark contrast to observations in BM.

Metabolite analysis of spleen from ATPIF1-KO mice revealed significant enrichment in metabolic pathways of glycolysis and amino acid synthesis. Notably, metabolites in glycolysis were elevated, and almost all amino acids were increased. Within the pentose phosphate pathway (PPP), level of D-sedoheptulose-7-phosphate was increased, which may indicate enhanced NADPH production. In addition, the accumulation of DHAP and glyceral-3-phosphate suggests upregulation of the glycerol synthesis pathway (Figure 5F). Increased ratios of ATP/ADP, lactate/pyruvate and malate/oxaloacetate indicate that the spleen is undergoing reductive stress (Xiao and Loscalzo, 2020). We speculate that the surge in demand for biomolecules and energy in cell proliferation helps to balance and mitigate the damage caused by reductive stress (Figures 5G,H). These data establish the spleen as a pivotal site for compensatory erythroid development in the context of ATPIF1 deficiency, sustaining erythroid homeostasis through coordinated metabolic and proliferative adaptations.

## Discussion

Our study unveils a previously unrecognized “tissue-compartmentalized metabolic adaptation” in mammalian erythropoiesis. Existing literature indicates that in cases of mitochondrial deficiency-related anemia, the spleen is unable to substitute the bone marrow in the production of red blood cells in patients (Menon et al., 2024). Our data suggest that ATPIF1 deficiency drives a divergent remodeling of mitochondrial function and metabolic flux between the BM and spleen, enabling the spleen to act as a “erythropoietic rescue niche” that mitigates severe anemia. The finding challenges two long-standing assumptions in the field: first, that ATPIF1’s role in erythropoiesis is conserved across vertebrates derived from the observation of non-compensatory severe anemia in ATPIF1-deficient zebrafish (Shah et al., 2012); and second, that splenic erythropoiesis only execute a stress-specific erythropoiesis and cannot replace BM erythropoietic programs (Menon et al., 2024). The finding reflects a mammalian-specific ability to rewire metabolic pathways in the spleen for erythropoiesis.

A core innovation of our work lies in identifying “reductive stress as a context-dependent regulator of erythroid fate” - a departure from the traditional focus on oxidative stress in erythropoietic dysfunction (Aragones et al., 2008). In the BM of ATPIF1-KO mice, reductive stress manifests as collapsed mitochondrial OXPHOS (evidenced by downregulated TCA cycle/OXPHOS genes, reduced ATP/ADP ratios, and depleted ROS), which creates a metabolic bottleneck for nucleotide biosynthesis and heme production. Specifically, the accumulation of 2-hydroxybutanoic acid disrupts NADH/NAD^+^ balance (a key redox couple for nucleotide synthesis), while diminished mitochondrial mass impairs heme biosynthesis despite elevated intracellular Fe^2+^. This decoupling of Fe^2+^ availability from heme production - previously unreported in ATPIF1-associated erythropoiesis - highlights a unique mechanism by which mitochondrial dysfunction disrupts iron-heme homeostasis in the BM.

In contrast, the spleen of ATPIF1-KO mice reconfigures reductive stress into a metabolic reprogramming that is distinct from both WT spleen and KO BM. Transcriptomic and metabolomic analysis reveal that splenic erythroid cells upregulate glycolysis (to compensate for OXPHOS defects) and the pentose phosphate pathway (PPP; marked by increased D-sedoheptulose-7-phosphate), which generates NADPH to buffer reductive stress through supporting nucleotide synthesis for proliferation. Critically, this reprogramming is accompanied by selective upregulation of heme biosynthesis genes (Fech and Hmbs), which is a regulatory step not observed in the BM. Fech catalyzes the final iron-incorporation step in heme synthesis, and Hmbs is a rate-limiting enzyme in porphyrin precursor production. They synergistically restore heme levels in the spleen despite ATPIF1 deficiency. This targeted activation of heme biosynthetic machinery, coupled with elevated Fe^2+^, explains why the spleen maintains heme homeostasis even as the BM fails, which represents a novel splenic-specific compensatory mechanism.

Our findings also redefine ATPIF1’s role in erythropoiesis beyond its canonical function as an ATP synthase regulator. While previous studies linked ATPIF1 deletion to mitochondrial proton gradient collapse and energy crisis (Acin-Perez et al., 2023), we demonstrate that ATPIF1 is additionally required for maintaining the metabolic flexibility of erythroid cells - i.e., their ability to switch between OXPHOS and glycolysis depending on tissue context. In the BM, ATPIF1 deficiency eliminates this flexibility, trapping erythroid cells in an energy-deficient state; in the spleen, metabolic flexibility is restored via glycolytic/PPP upregulation, suggesting that ATPIF1’s absence can be bypassed if alternative metabolic pathways are activated. This challenges the view that ATPIF1 is an indispensable regulator of erythroid mitochondria and instead positions it as a “metabolic checkpoint” that modulates pathway selection.

Clinically, our work opens new avenues for treating mitochondrial-related anemias (e.g., those associated with Complex V defects or inherited ATPIF1 variants). The observation that splenic erythroid cells can overcome ATPIF1 deficiency via glycolytic/PPP activation suggests that pharmacological agents targeting these pathways (e.g., PPP activators like metformin or glycolytic enhancers) could mitigate anemia in patients with mitochondrial dysfunction. Additionally, the spleen’s ability to restore heme homeostasis via Fech/Hmbs upregulation identifies these genes as potential therapeutic targets, for example, via gene editing or small-molecule activators to bypass BM heme synthesis defects.

A critical unresolved question is the role of the splenic microenvironment in driving this compensatory adaptation. The spleen’s niche (composed of stromal cells, macrophages, and endothelial cells) likely secretes cytokines or growth factors that trigger metabolic reprogramming in erythroid progenitors for cell proliferation. For instance, splenic macrophages may enhance iron recycling (via ferroportin upregulation) to support Fe^2+^ availability, while stromal cells could secrete SCF or EPO to promote proliferation. Additionally, spleen recycling of heme from senescent red blood cells may contribute to the mechanism of tissue-specific erythropoiesis. This recycling activity is not available in the bone marrow, which may contribute to increased cell death from the reductive stress. Like the oxidative stress, reductive stress is able to induce cell death (Ge et al., 2024). Future co-culture experiments will clarify how non-erythroid cells in the spleen create a permissive niche for ATPIF1-deficient erythroid cells, which may address broad implications for understanding extramedullary hematopoiesis in other mitochondrial disorders (Menon et al., 2024).

In summary, our study advances the field by: (1) identifying a tissue-specific metabolic adaptation to ATPIF1 deficiency that distinguishes mammals from lower vertebrates; (2) uncovering reductive stress as a context-dependent regulator of erythroid survival and differentiation; (3) defining a novel splenic mechanism for restoring heme homeostasis via Fech/Hmbs upregulation; and (4) repositioning ATPIF1 as a metabolic flexibility regulator rather than a strict requirement for erythropoiesis. These insights provide new insight into mitochondrial-erythroid interactions but also provide a framework for developing targeted therapies for anemias linked to mitochondrial dysfunction.

## Data Availability

The datasets presented in this study can be found in online repositories. The names of the repository/repositories and accession number(s) can be found below: https://www.ncbi.nlm.nih.gov/, SUB15446929 https://www.ncbi.nlm.nih.gov/, SUB15458451 https://www.ebi.ac.uk/metabolights/, MTBLS13085.
