# Supplementary material for: Splenic compensation alleviates impaired-development of bone marrow terminal erythroid to attenuate anemia in ATPIF1 knockout mice
Source: Front Cell Dev Biol. 2025 Oct 17;13:1675547. doi: 10.3389/fcell.2025.1675547 (PMC12575250; doi:10.3389/fcell.2025.1675547)
Supplement: Supplementary file 1 [file DataSheet1.docx]

Supplement

Table. Blood parameters of WT and ATPIF1-KO mice.

| Parameters | WT | ATPIF1-KO |
| --- | --- | --- |
| RBC (*10^12 cells/L) | 9.59±0.38 | 7.94±0.36* |
| HGB (g/L) | 145.5±9.68 | 111.7±4.34** |
| HCT (%) | 42.82±2.88 | 35.23±1.49* |
| MCV (fL) | 44.55±1.81 | 44.53±0.99 |
| MCH (pg) | 15.18±0.90 | 14.83±0.55 |
| MCHC (g/L) | 347.3±32.42 | 336.5±19.81 |
| WBC (*10^9 cells/L) | 5.45±0.60 | 5.16±0.71 |
| Lymph# (*10^9 cells/L) | 5.00±0.58 | 4.38±0.61 |
| Gran# (*10^9 cells/L) | 0.7±0.03 | 0.85±0.12 |


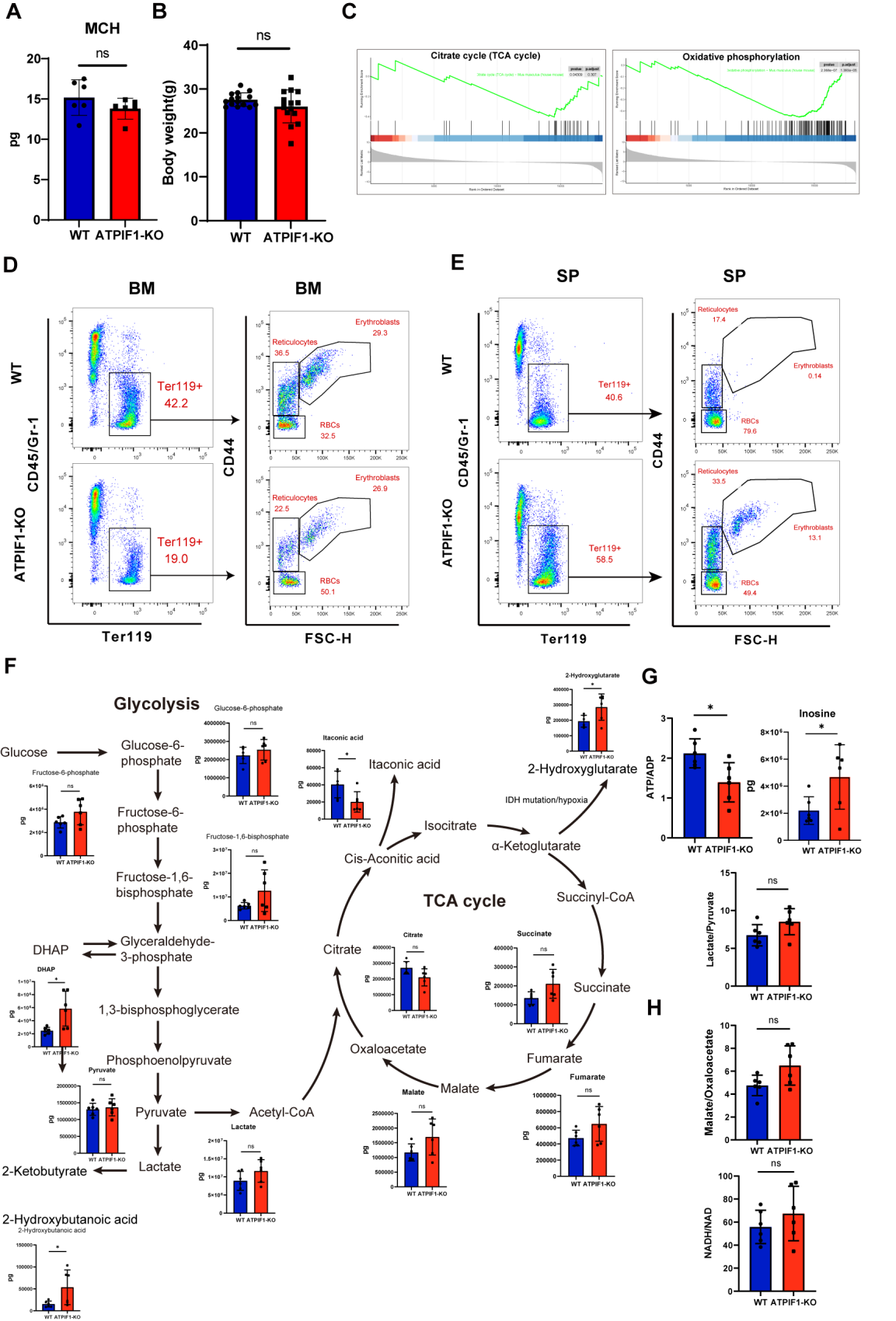


Figure 1. GSEA and metabolic characterization of bone marrow in ATPIF1-KO mice.

(A) Mean corpuscular hemoglobin (MCH) levels in peripheral blood (n=6). (B) Body weight analysis (n=15). (C) GSEA of RNA-seq data revealing downregulation of TCA cycle and OXPHOS pathways in BM CD45^-^ cells, ATPIF1-KO is indicated in blue. (D) Flow cytometric analysis of BM erythroid populations (Ter119^+^/CD45^-^/Gr-1^-^) gated by CD44 expression levels into erythroblasts, reticulocytes and RBCs (n=24). (E) Flow cytometric analysis of erythroid populations (Ter119^+^/CD45^-^/Gr-1^-^) gated by CD44 expression levels into erythroblasts, reticulocytes and RBCs in the spleen (n=24). (F) Alterations of bone marrow metabolites in glycolysis and the TCA cycle. (G) Alterations in ATP/ADP, Inosine and Lactate/Pyruvate ratios in bone marrow (n=6). (H) Alterations in Malate/Oxaloacetate and NADH/NAD ratios in bone marrow (n=6). Data are presented as mean ± SD; ns>0.05; * *p*<0.05; ** *p*<0.01; *** *p*<0.001 by unpaired two-tailed t test, NES and FDR-adjusted p values by clusterProfiler are shown (C).


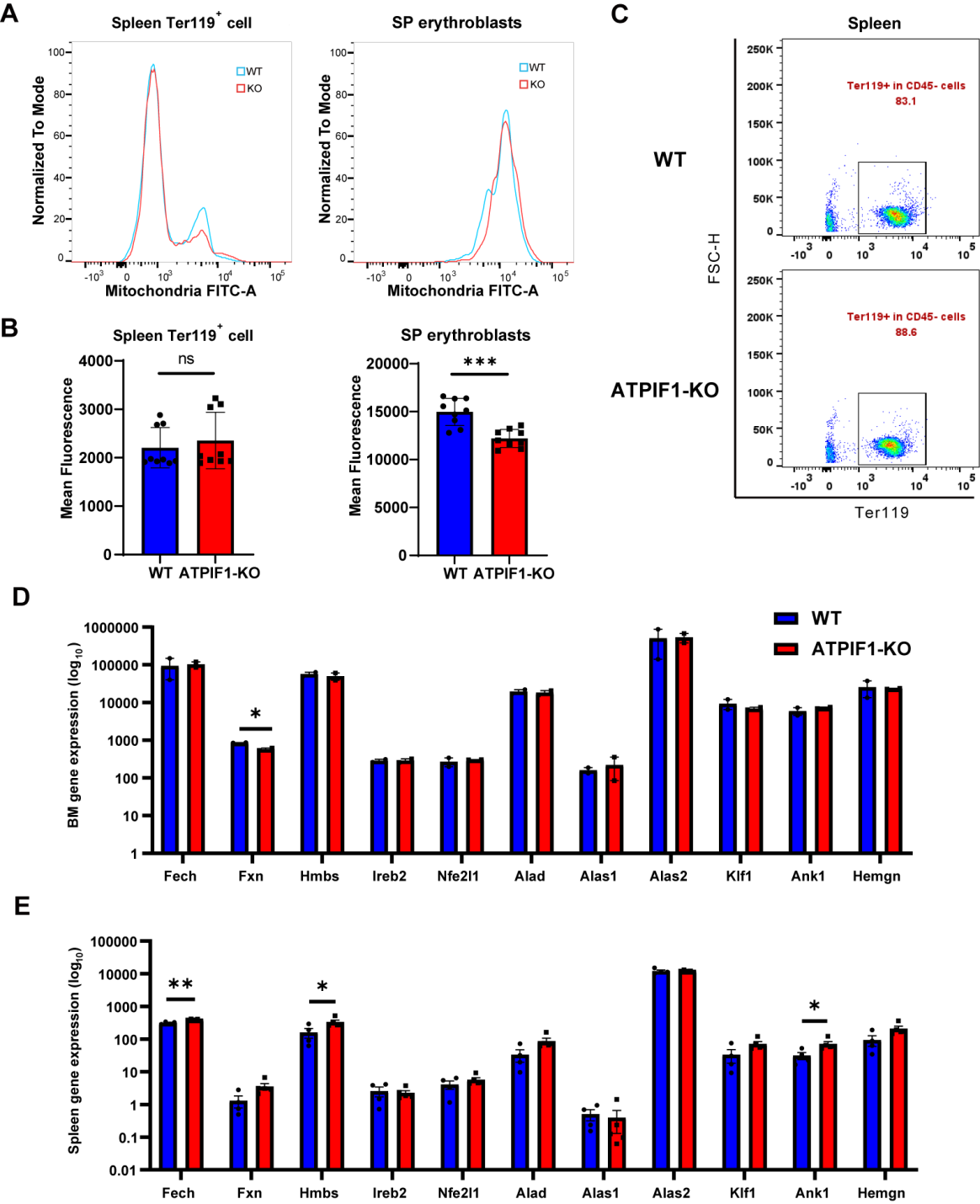


Figure 2. Alterations in mitochondrial and iron metabolism–related genes in ATPIF1-KO mice.

(A) Representative flow cytometry profiles illustrating mitochondrial content, assessed by MitoTracker Green staining, in splenic Ter119^+^ cells and erythroblast populations. (B) Quantitative analysis of mitochondrial fluorescence intensity in distinct splenic erythroid cells subpopulations (n=9). (C) Proportion of Ter119^+^ cells among CD45^-^ spleen cells following magnetic bead sorting. (D) Log10-transformed gene expression levels of heme biosynthesis-associated genes in bone marrow (n=2). (E) Log10-transformed expression levels of heme biosynthesis-associated genes in spleen (WT:ATPIF1-KO=4:5). Data are presented as mean ± SD; ns>0.05; * *p*<0.05; ** *p*<0.01; *** *p*<0.001 by unpaired two-tailed t test.
